# Vestibular Schwannoma: Long-term Outcome of the Vestibular Function After Stereotactic Radiosurgery

**DOI:** 10.1097/ONO.0000000000000038

**Published:** 2023-09-12

**Authors:** Lukas Anschuetz, Ekin Ermiş, Isabel Gebhart, Odile Stalder, Andreas Raabe, Georgios Mantokoudis, Marco Caversaccio, Evelyne Hermann, Franca Wagner, Dominique Vibert

**Affiliations:** 1 Department of Otorhinolaryngology, Head & Neck Surgery, Inselspital, University Hospital and University of Bern, Bern, Switzerland; 2 Department of Radiation-Oncology, Inselspital, Bern University Hospital and University of Bern, Bern, Switzerland; 3 CTU Bern, University of Bern, Bern, Switzerland; 4 Department of Neurosurgery, Inselspital, University Hospital and University of Bern, Bern, Switzerland; 5 Department of Neuroradiology, Inselspital, University Hospital and University of Bern, Bern, Switzerland.

**Keywords:** Vestibular schwannoma, Stereotactic radiosurgery, Vestibular function, Videonystagmography, c-VEMPS, v-HIT

## Abstract

**Objective:**

Evaluation at long term of the impact of the stereotactic surgery (SRS) on the vestibular function in vestibular schwannoma (VS) patients.

**Study design and setting:**

Retrospective study in a tertiary referral center.

**Patients:**

Fifty-one VS patients were included (34 females;17 males), aged from 41 to 78 years treated exclusively with SRS.

**Intervention:**

Vestibular function was assessed before SRS and with median time interval of 14 (FU1) and 25 (FU2) months after treatment. Vestibular evaluation included: history, clinical vestibular examination, videonystagmography, head impulse test (v-HIT) and cervical vestibular evoked myogenic potentials (c-VEMPS).

**Results:**

Before SRS, caloric testing (Caloric) was impaired in 77%; after treatment, in 92% (FU1) and 77% (FU2). Lateral HIT was decreased in 22% before SRS, in 39% at FU1 and FU2. C-VEMPS were absent in 50% before SRS, in 76% at FU1 and, FU2. Before SRS, no statistically significant association was found between asymptomatic and symptomatic patients with respect to the results of Caloric, v-HIT and c-VEMPS. This lack of association was also seen after SRS, at FU1 and FU2.

**Conclusion:**

Our study showed that the impairment of the vestibular function might be attributed to the VS itself as well as to the radiation of the inner ear during SRS. The lateral SSC at low frequencies and the saccular function seem to be more involved with the time.

## INTRODUCTION

Vestibular schwannoma (VS) is a benign tumor of the central nervous system, arising from the vestibular branch of the eighth cranial nerve. It represents 6% of all intracranial tumors (1). Due to its slow growth, VS can remain asymptomatic. Nevertheless, the key symptoms are progressive hearing loss, sudden deafness, and tinnitus (2,3). In the literature, vertigo such as sensation of spinning and postural symptoms such as unsteadiness was also reported in half of patients (2,4). In 5%–18% of patients, vestibular symptoms can mimic the typical triade of Meniere’s disease (4–6) but are reported as shorter and of low to moderate intensity compared to Meniere’s attacks (4).

The progressive growth of VS, from the IAC through the cerebellopontine angle, may lead to a compression of the cochleo-vestibular nerve, the adjacent nerves, particularly the V and VII nerves, and the brainstem with the associated neurological symptomatology. Depending on VS size and associated symptoms, 3 management strategies are available. The “wait-and-scan” option is recommended in small VS size, asymptomatic or with very few cochleo-vestibular symptoms. For symptomatic tumors larger than 2 cm, and symptoms related to brainstem compression by mass effect, the surgical option represents the treatment of choice (7). Microsurgical removal allows a good control on VS growth but may be associated to several and not rare intra- and postoperative complications (8). First described in 1971 by Leksell (9), radiation of VS has been shown to represent a safe alternative to the microsurgery. Thus, since several years, stereotactic radiosurgery (SRS) confirmed its efficacy to control tumor growth (10–12). SRS is recommended for small to moderate sized, newly diagnosed VS or for growing VS without mass effect (13).

Although the assessment of hearing after SRS has been extensively investigated, this is not the case for the vestibular function. To date, the objective outcome of the vestibular function is rarely reported in the literature (14,15).

The aim of our study was to report the long-term outcome of the vestibular function in patients who underwent SRS, as initial treatment. We assume as main hypothesis, that like for hearing, the SRS might have a strong impact on the vestibular function, at short but maybe also at long term after SRS treatment.

## PATIENTS AND METHODS

This retrospective study was approved by the Ethics Committee of the Canton of Bern (KEK-BE: 2017-02127) and performed in accordance with the Declaration of Helsinki (16). We reviewed the medical charts of all patients with VS, who were treated with SRS, at our institution between 2009 and 2018. Inclusion criteria were adult patients, exclusively treated with SRS for unilateral, sporadic VS and who underwent a neurotological examination before SRS. Patients with prior surgery or fractioned irradiation as well as patients without neurotological examination before SRS, were excluded from the study.

### Subjective and Objective Evaluation of the Vestibular Function

In our institution, all VS patients, candidates to SRS, with and without vestibular symptoms, underwent a neurotological examination before treatment including medical history, clinical vestibular examination (CVE) with assessment of the vestibulo-spinal reflex, using Romberg and Unterberger’s stepping tests. History pointed out the presence or absence of episodic vertigo, and unsteadiness, such as defined by Bisdorff et al (17).

Neurotological examination (ONO) included audiogram, auditory evoked brainstem potentials (Eclipse EP25, Interacoustics, Denmark). Videonystagmography (VNG) (VNG NysStar II, Difra Instrumentation, Belgium) recorded spontaneous and positional nystagmus. Vestibulo-ocular reflex (VOR) was measured using the rotatory pendular test, including visual fixation suppression and the bithermal caloric test (Caloric) with ice-water irrigation if needed. The function of the 6 semicircular canals (SCC) at high frequencies was assessed using the video head impulse test (v-HIT) (ICS Impulse, Otometrics, Denmark). The sacculocollic reflex (SCR) was recorded using the click-evoked cervical vestibular evoked myogenic potentials (c-VEMPS) (Eclipse VEMP, Intercoustics GmbH, Germany).

At our laboratory, we defined an areflexia of the lateral SCC at Caloric as an absence of nystagmic response and a hyporeflexia as an unilateral weakness >20%. Gains of VOR <0.8 (for horizontal canals) and <0.6 (for vertical canals) were considered as decreased at v-HIT. Within c-VEMPS, the presence or absence of P1 and N1 determined the presence or absence of SCR, respectively.

Neurotological examinations were performed at different time delays after SRS. In our study, we included all patients who underwent neurotological examinations with a long-term FU, 1 and 2 years after SRS.

### Neuroradiological Imaging Analysis

Evaluation of MRI scans was performed by our experienced board-certified neuroradiologist (FW). The tumor size was graded before SRS according to the following institutionally modified Koos Classification (18): *Koos stage 1* (tumor confined to the internal auditory canal (IAC)); *stage 2* (extension over the IAC without contact to the brainstem); *stage 3* (contact with the brainstem without compression); *stage 4a* (compression of the brainstem without infratentorial midline shift); *stage 4b* (compression of the brainstem with infratentorial midline shift).

Measurement of the tumor volume was performed by FW using 3D Slicer 4.4.0 (freely downloadable from the website https://www.slicer.org). 3D Slicer software was used to get a better accuracy and quality support. Briefly, it is a personal-computer-based image analysis tool where the region of interest (VS) is manually contoured in each slice. The software then calculates the volume of the contoured region, and the total volume is reported in cubic millimeters. For all patients, we used the contrast-enhanced 3D T1w multiplanar reconstruction (MPR) sequence for the delineation of the tumor mass.

### Stereotactic Irradiation

Tumors were treated using LINAC (Novalis, BrainLAB, Munich, Germany) and robotic (Cyberknife, Accuray; Sunnyvale, USA) based SRS. Patients were immobilized in supine position on the treatment table, using a commercial stereotactic mask fixation system. The iPlan (Brain LAB, Munich, Germany) and Multiplan (Accuray, Sunnyvale, USA) treatment planning system were used to generate radiosurgery plans. Target volumes were delineated slice by slice in axial view, using postcontrast thin-slice (1 mm) gadolinium-enhanced T1- and T2-weighted axial magnet resonance imaging (MRI) sequences fused with thin-slice (0.75 mm) planning computed tomography scans. Target definition and dose prescriptions were based on international consensus guidelines (13). A single fraction of 12 Gy with a mean prescription isodose line of 94% (range, 85–99) and 78% (range, 52–90) was prescribed for the Novalis and Cyberknife systems, respectively.

### Statistical Analysis

Stata 17 (Stata Corp., College Station, Texas) was used to perform the statistical analyses. Descriptive statistics were used to investigate vestibular symptoms and function in patients’ over time. Due to the fact that the FU were not performed at exactly regular intervals, we introduced time intervals 10–18 months (FU1), 19–30 months (FU2) and analyzed the most centered follow-up (FU) record per patient. The association between tumor size and dizziness before irradiation and overtime since irradiation was investigated using linear or logistic mixed-effects models as appropriate. Lateral SCC function was measured using Caloric (Jongkee’s formula) and reported as episodic vertigo and permanent unsteadiness as binary variables (ie, present/absent).

Association, before SRS, between the Koos stages of VS and volumetry (in cubic centimeters), and versus the values of Caloric ipsilateral to VS were investigated though logistic or linear models. The same models were used to investigate the association between the volumetry versus the Caloric. Vestibular tests results were compared between symptomatic and asymptomatic patients using Chi-squared test and the non-parametric Wilcoxon rank-sum test as appropriate. The vestibular symptoms associated with ipsilateral Caloric, c-VEMPS, v-HIT were also investigated through logistic models. A *P* value <0.05 was considered as statistically significant.

## RESULTS

Fifty-one patients fulfilled the criteria of inclusion: 27 males and 24 females aged from 41 to 78 years old (mean age: 63 years). VS was on the left side in 34 patients. According to the modified Koos classification (18), VS were graded as: stage 1 (n = 10), stage 2 (n = 24), stage 3 (n = 8), and stage 4a (n = 9).

Before SRS, clinical vestibular (CVE) and neurotological examinations (ONO) were performed within all 51 patients. After SRS, from those patients, 40 patients underwent both CVE and ONO at FU1 (10–18 (mean: 14) months). From these 40 patients, CVE was performed within 31 patients, and ONO within 30 patients, at FU2 (19–30 (mean: 25) months).

### Clinical Vestibular Examination

Table 1 summarizes the symptoms and clinical findings before and after SRS.

**TABLE 1.** Symptoms and clinical findings at CVE before and after SRS

	Before SRS	FU1	FU2
	N = 51	N = 40	N = 31
**Episodic vertigo**	9 (17%)	2 (5%)	1 (3%)
**Unsteadiness**	6 (12%)	10 (40%)	3 (10%)
**SpontaneousNystagmus**	6 (11%)	9 (23%)	4 (13%)
Grade I	3	2	1
Grade II	2	4	2
Grade III	1	3	1
**Romberg test**			
Normal	44 (86%)	34 (85%)	28 (90%)
Deviation ipsilateral VS	5 (10%)	2 (5%)	3 (10%)
Deviation both side	2 (4%)	4 (10%)	
**Unterberger test**			
Normal	35 (69%)	23 (57%)	18 (58%)
Deviation ipsilateral VS	13 (26%)	15 (38%)	12 (39%)
Deviation both side	3 (5%)	2 (5%)	1 (3%)

CVE indicates clinical vestibular examination; SRS, stereotactic surgery; VS, vestibular schwannoma.

Before SRS, 15 patients were symptomatic. From those, 4 patients (27%) remained symptomatic at FU2.

From the 36 asymptomatic patients before SRS, 8 patients (40%) complained of vestibular symptoms at FU1. At FU2, all patients were asymptomatic again.

Statistically, there was no significant association between the VS size at baseline and CEV before SRS and overtime (table 2). It was the case for vestibular symptoms such as unsteadiness and episodic vertigo as well as for the clinical findings such as spontaneous nystagmus and Romberg test. The association was slightly significant for Unterberger test.

**TABLE 2.** Association between the VS size at baseline versus the CVE and the ONO, before SRS and overtime after SRS

	Before SRS		Overtime	
CVE	OR (95% CI)	*P*	OR (95% CI)	*P*
Unsteadiness	1.006 (0.835 to 1.212)	0.949	1.092 (0.929 to 1.282)	0.286
Episodic vertigo	1.072 (0.934 to 1.230)	0.325	1.050 (0.977 to 1.129)	0.185
Spontaneous nystagmus	1.110 (0.944 to 1.305)	0.205	1.124 (0.971 to 1.300)	0.118
Romberg test	1.010 (0.986 to 1.034)	0.407	1.008 (0.990 to 1.027)	0.382
Unterberger test	1.191 (1.001 to 1.416)	**0.049**	1.168 (1.011 to 1.350)	**0.036**
**ONO tests**				
Ipsilateral caloric	0.881 (–13.095 to 14.858)	0.902	0.995 (–12.006 to 13.996)	0.881
Ipsilateral v-HIT				
Lateral SCC	0.969 (0.808 to 1.163)	0.739	0.997 (0.838 to 1.186)	0.973
Anterior SCC	1.010 (0.762 to 1.338)	0.947	0.978 (0.753 to 1.270)	0.865
Posterior SCC	0.814 (0.628 to 1.056)	0.121	0.908 (0.731 to 1.127)	0.381
c-VEMPS ipsilateral	1.021 (0.819 to 1.272)	0.856	0.961 (0.816 to 1.132)	0.636
c-VEMPS contralateral	0.940 (0.749 to 1.179)	0.593	0.884 (0.736 to 1.061)	0.185

Ant indicates anterior SCC; C-VEMPS, cervical vestibular evoked myogenic potentials; CVE, clinical vestibular examination; HIT, head impulse test; Lat, lateral SCC; ONO, neurotological examination; post, posterior SCC; SCC, semicircular canals; SRS, stereotactic surgery; VS, vestibular schwannoma; vHIT, video head impulse test.

### Neurotological Examination

**FIG. 1.**
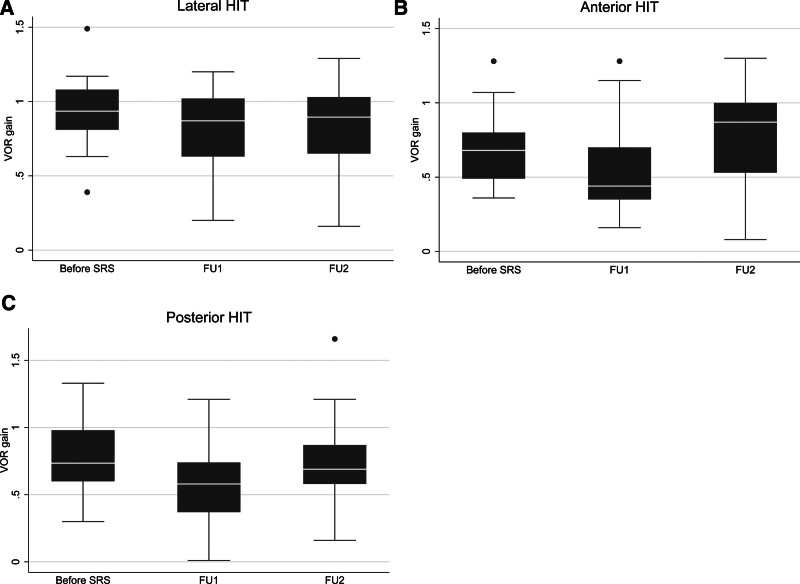
VOR gain results at v-HIT, for the lateral (*A*), anterior (*B*), and posterior (*C*) SCC. Box plots depicting the medians and the IQR of VOR gain before SRS, at FU1 and FU2. The circles indicate outliers. *A*, Values of medians (IQR): 0.94 (0.81; 1.1), 0.87 (0.63; 1.0), 0.89 (0.65; 1.0); *B*, Values of medians (IQR): 0.68 (0.49; 0.80), 0.44 (0.35; 0.70), 0.87 (0.53;1.0); *C*: Values of medians (IQR): 0.74 (0.60; 0.98), 0.58 (0.37; 0.74), 0.69 (0.58; 0.87). IQR indicates interquartile range.

Table 3 details the findings for lateral SCC and saccular functions assessed with Caloric, lat-HIT and SCR ipsi and contralateral to VS, before SRS, at FU1 and FU2. Figure 1 illustrates the VOR gain of the lateral, anterior, and posterior SCC, ipsilateral to VS, before SRS, at FU1 and FU2.

**TABLE 3.** Aggregate results of lateral SCC and saccular functions, ipsi and contralateral to VS, before, and overtime after SRS

	Before SRS	FU1	FU2	*P*
Caloric	N = 51	N = 40	N = 30	
**Ipsilateral**				**0.004**
Normal	12 (23%)	3 (8%)	4 (13%)	
Hyporeflexia	29 (57%)	18 (45%)	9 (30%)	
Areflexia	10 (20%)	19 (47%)	17 (57%)	
**Contralateral**				**0.012**
Normal	51 (100%)	36 (90%)	26 (87%)	
Hyporeflexia	0	4 (10%)	4 (13%)	
Areflexia	0	0	0	
**Lat-HIT**	**N = 18**	**N = 23**	**N = 18**	
**Ipsilateral**				0.456
Normal	14 (78%)	14 (61%)	11 (61%)	
Decreased	4 (22%)	9 (39%)	7 (39%)	
**Contralateral**				0.758
Normal	17 (94%)	22 (96%)	17 (94%)	
Decreased	1 (6%)	1 (4%)	1 (6%)	
**c-VEMPS**	**N = 32**	**N = 34**	**N = 25**	
**Ipsilateral**				**0.046**
Present	16 (50%)	8 (24%)	6 (24%)	
Absent	16 (50%)	26 (76%)	19 (76%)	
**Contralateral**				0.707
Present	22 (69%)	20 (59%)	15 (60%)	
Absent	10 (31%)	14 (41%)	10 (40%)	

Fisher exact test was used. A *P* value <0.05 was set as statistically significant.

c-VEMPS indicates cervical vestibular evoked myogenic potentials; Lat-HIT, head impulse test for the lateral semi-circular canal; N, the number of patients for each vestibular test before SRS and at FU1 and FU2.

#### Caloric Ipsilateral to VS

Caloric ipsilateral to VS was impaired within 77% of patients before SRS, in 92% of patients at FU1, and in 87% at FU2. A contralateral hyporeflexia was measured after SRS in 10% and 13% of patients at FU1 and FU2, respectively.

Statistically, there was no significant association between Caloric values versus volumetry (association coefficient 1.14, *P* = 0.887) and versus the stages of Koos classification (OR 1.00, *P* = 0.814) before SRS. VS volume and Caloric values were also not significantly associated overtime (Table 2).

#### v-HIT

VOR gain of the lateral SCC ipsilateral to VS was decreased within 22% of patients before SRS. This percentage reached 39% at FU1 and FU2.

Statistically, there was no significant association between VS volume and ipsilateral v-HIT before SRS: for lateral, anterior, and posterior SCC as well as overtime (Table 2).

#### c-VEMPS

c-VEMPS ipsilateral to VS were absent within 50% of patients before SRS, in 76% at FU1 and FU2. Contralateral to VS, SCR was absent within 31% of patients before SRS. This percentage reached 41% and 40% at FU1 and FU2, respectively.

Again, statistically there was no significant association between VS volume and c-VEMPS ipsilateral and contralateral to VS before SRS and overtime (Table 2).

In summary, the impairment of the lateral SCC function, ipsilateral to VS, was statistically significant for the low frequencies of VOR after SRS (*P =* 0.004). Surprisingly, a hypofunction was also statistically significant, contralateral to VS, overtime (*P* = 0.012). The saccular function ipsilateral to VS was also significantly impaired after SRS (*P* = 0.046).

### Saccular and SCC Functions Within Symptomatic and Asymptomatic Patients Before SRS and Overtime

Table 4 compares the vestibular tests results between the symptomatic and asymptomatic patients for the saccular and SCC functions ipsilateral to VS, before SRS, at FU1 and FU2. No statistically significance was found between both groups.

**TABLE 4.** Comparison of vestibular tests for asymptomatic and symptomatic patients before, at FU1 and FU2 after SRS

	All patients n (%)	Asymptomatic patients n (%)	Symptomatic patients n (%)	*P*
**Before SRS**	**N = 51**			
**Caloric**	**N = 51**	**N = 36**	**N = 15**	0.77
Normal	12 (23%)	9 (25%)	3 (20%)	
Hyporeflexia	29 (57%)	19 (53%)	10 (67%)	
Areflexia	10 (20%)	8 (22%)	2 (13%)	
**Lateral HIT**	**N = 18**	**N = 13**	**N = 5**	1.00
Normal	14 (78%)	10 (77%)	4 (80%)	
Decreased	4 (22%)	3 (23%)	1 (20%)	
**Anterior HIT**	**N = 17**	**N = 12**	**N = 5**	1.00
Normal	10 (59%)	7 (58%)	3 (60%)	
Decreased	7 (41%)	5 (42%)	2 (40%)	
**Posterior HIT**	**N = 18**	**N = 13**	**N = 5**	1.00
Normal	14 (78%)	10 (77%)	4 (80%)	
Decreased	4 (22%)	3 (23%)	1 (20%)	
**c-VEMPS**	**N = 32**	**N = 20**	**N = 12**	1.00
Absent	16 (50%)	9 (47%)	6 (50%)	
Present	16 (50%)	11 (53%)	6 (50%)	
**FU1**	**N = 40**			
**Caloric**	**N = 40**	**N = 12**	**N = 28**	0.28
Normal	3 (8%)	0 (0%)	3 (11%)	
Hyporeflexia	18 (45%)	4 (33%)	14 (50%)	
Areflexia	19 (47%)	8 (67%)	11 (39%)	
**Lateral HIT**	**N = 23**	**N = 8**	**N = 15**	0.18
Normal	14 (61%)	3 (37%)	11 (73%)	
Decreased	9 (39%)	5 (63%)	4 (27%)	
**Anterior HIT**	**N = 21**	**N = 7**	**N = 14**	0.047
Normal	7 (33%)	0 (0%)	7 (50%)	
Decreased	14 (67%)	7 (100%)	7 (50%)	
**Posterior HIT**	**N = 22**	**N = 7**	**N = 15**	1.00
Normal	10 (45%)	3 (43%)	7 (47%)	
Decreased	12 (55%)	4 (57%)	8 (53%)	
**c-VEMPS**	**N = 34**	**N = 9**	**N = 25**	0.65
Absent	26 (76%)	6 (67%)	20 (80%)	
Present	8 (24%)	3 (33%)	5 (20%)	
**FU2**	**N = 30**			
**Caloric**	**N = 30**	**N = 11**	**N = 19**	0.18
Normal	4 (13%)	0 (0%)	4 (21%)	
Hyporeflexia	9 (30%)	5 (45%)	4 (21%)	
Areflexia	17 (57%)	6 (55%)	11 (58%)	
**Lateral HIT**	**N = 18**	**N = 7**	**N = 11**	1.00
Normal	11 (61%)	4 (57%)	7 (64%)	
Decreased	7 (39%)	3 (43%)	4 (36%)	
**Anterior HIT**	**N = 17**	**N = 7**	**N = 10**	0.59
Normal	12 (71%)	4 (57%)	8 (80%)	
Decreased	5 (29%)	3 (43%)	2 (20%)	
**Posterior HIT**	**N = 15**	**N = 7**	**N = 5**	0.61
Normal	10 (67%)	4 (57%)	6 (75%)	
Decreased	5 (33%)	3 (43%)	1 (25%)	
**c-VEMPS**	**N = 25**	**N = 8**	**N = 17**	0.62
Absent	19 (76%)	7 (88%)	12 (71%)	
Present	6 (24%)	1 (12%)	5 (29%)	

Chi-squared test was used. A *P* value <0.05 was set as statistically significant.

Ant indicates anterior SCC; C-VEMPS, cervical vestibular evoked myogenic potentials; HIT, head impulse test; Lat, lateral SCC; Post, posterior SCC; SRS, stereotactic surgery.

## DISCUSSION

Our study showed an impairment or an aggravation of the lateral SCC and saccular functions at long-term after SRS. A high number of studies described the effects of VS and irradiation on the cochlea and the cochlear nerve. However, it is not the case for the vestibular part of the VIII nerve. We would firstly like to discuss the literature regarding mechanisms of hearing loss in VS, which is usually attributed to a dysfunction of the cochlear nerve, dysfunction of the cochlea itself, or both (19). Histopathologic findings demonstrated atrophy of the cochlear nerve (20,21). High-dose of radiation on the inner ear (nasopharyngeal cancer treatment) is known to be ototoxic with dose-dependent histopathological changes such as atrophy of the stria vascularis, stria ligament as well as decreased outer and inner hair cells (22,23). In contrast, the retro-cochlear auditory pathways remain functionally intact even after radiation doses from 24 to 62 Gy delivered to the cochlea and IAC (24).

In SRS treatment, lower dose of radiation (12Gy) was recommended to treat VS (13). Nevertheless, Linskey et al (25) reported unintentionally higher dose than 12 Gy in the basal turn of the cochlea near the modiulus as well as in the vestibular labyrinth particularly at the ampulated ends of the lateral and posterior SCC. More rarely, higher dose was also detected at the endolymphatic sac. Preservation of serviceable hearing depends of several criteria, including the volume of the cochlea. Kano et al (26) demonstrated a significantly better hearing preservation if the dose radiation delivered on the central cochlea was <4.2 Gy. Nevertheless, 10 years post SRS, several studies showed that the hearing continued to decline (27). Recently, Ermis et al (28) found that radiation with 5 Gy or more on the vestibule contributed to increase significantly the symptomatology of dizziness after SRS.

Regarding the vestibular symptoms, our study showed that, before SRS, a third of patients complained of recurrent episodic vertigo or permanent unsteadiness. This percentage is lower than that reported by Kentala and Pyykkö (4) probably because our cohort of patients was focused on VS eligible for SRS treatment. Indeed, our percentage of symptomatic patients is like that of BojrabII et al (29), who reported vestibular symptoms in 34.7% of patients before SRS. In our study, 1 year after SRS, the percentage of symptomatic patients increased to 45%, and reached always 13% 2 years, after treatment. Thus, we attributed the high percentage of vestibular symptoms, as inherent to the radiosurgery itself (19,25). Two hypotheses might explain these findings: firstly, a lack of central compensation of the vestibular deficit induced by the radiation and secondly a continuous decline of the vestibular function, as it was observed for the hearing function (27). In our institution, to reach an optimal central compensation of the vestibular deficit after SRS, all symptomatic patients participated in an intensive vestibular rehabilitation program consisting of daily exercises (30), reinforced with weekly sessions of instrumented rehabilitation including dynamic posturography and optokinetic stimulation. The symptomatic patients performed this program of rehabilitation with success during the first weeks to 18 months after SRS. Nevertheless, despite of regular daily vestibular exercises and weekly sessions, 4 patients who complained of vertigo before SRS, remained symptomatic 2 years after treatment. Thus, the presence of symptoms before SRS appeared to compromise the prognosis in terms of central compensation at long term.

The fact that their contralateral saccular and SCC functions remained normal, may exclude a mechanism of compensatory down-regulation to explain their symptomatology. However, a progressive impairment of the vestibular labyrinth due to the VS itself, aggravated by the radiation might play a role. Nonetheless, others factors have also to be considered such as visual deficits, neurological diseases, as well as medications interfering in the central vestibular compensation, thus, in the balance control.

Regarding the lateral SCC function at low frequencies, we found, before SRS, a high percentage of weakness to absence of caloric response ipsilateral to VS, according to the literature (31–33). One year after SRS, the weakness remained present and tended to worsen, with an increasing number of areflexia overtime. As for the hearing function, these findings might reinforce the hypothesis of a continuing deterioration of the vestibular function after SRS.

In contrast, at high frequencies, the lateral SCC function remained normal within most of patients, before SRS. This discrepancy of results between low and high frequencies was also reported in the literature (34–36). In our study, at long-term FU, we observed the same discrepancy between VOR at high and low frequencies. Unlike the study of Lee et al (37), our results showed normal VOR gain at high frequencies, contralateral to the VS before and at all stages of FU. This difference might be explained by the fact that their patients’ cohort was different, in terms of tumor size and number of patients. In the literature, the impairment of the VOR contralateral to the lesioned side in case of VS, but also after vestibular surgical deafferentation or vestibular neuritis, is well known but poorly understood (37). Several theories were proposed, including a mechanism of compensatory down-regulation via commissural connections between both vestibular nuclei (38,39).

Regarding the saccular function, like others authors (37,40) but less than West et al (36), we found an absence of SCR in half of patients before SRS. After SRS, this percentage increased to 76%. Surprisingly, contralateral to VS, the SCR was absent in a third of our patients before SRS and this percentage increased after treatment. Knowing that VS grows slowly and is originated from the superior or inferior part of the vestibular nerve, one can hypothesize a similar mechanism of compensatory down-regulation via the commissural pathways to explain the impairment of the contralateral SCR (41). Finally, like Kentala and Pyykkö (4), at baseline, we found no association between asymptomatic and symptomatic patients versus SCC and saccular functions. It was also the case overtime after SRS.

Our study does have several limitations due to its retrospective design. First, during the last decade, new electrophysiological methods were developed to assess the function of the SCC and the otolithic organs. Then, it was not possible to collect the results of all examinations such as v-HIT and c-VEMPS for each patient. Second, we did not have a control group of VS patients, such as patients belonging to the “wait-and-scan” option. Further prospective studies should be performed to compare the vestibular function of both groups. It should provide insight into the natural history of the evolution of the vestibular function versus the impact of SRS on it.

## CONCLUSIONS

Our study showed that the impairment of the vestibular function may be attributed to the VS itself as well as to the radiation of the inner ear during SRS. The lateral SCC, at low frequencies, as well as the saccular functions seem to be more involved and aggravated with the time. Further prospective studies are needed to confirm these findings. Furthermore, we encourage the assessment of the vestibular function systematically before SRS, including an intensive vestibular rehabilitation within symptomatic patients before treatment as well as after SRS.

## FUNDING SOURCES

None declared.

## CONFLICT OF INTEREST

LA is a consultant for Stryker ENT.

## DATA AVAILABILITY STATEMENT

The datasets generated during and/or analyzed during the current study are not available.
